# A Novel Two-Dimensional Hydrophone Based on Fiber Bragg Gratings

**DOI:** 10.3390/s26051605

**Published:** 2026-03-04

**Authors:** I-Nan Chang, Wei-Chen Li, Chang-Chun Kuo, Wen-Fung Liu

**Affiliations:** 1 Department of Electronic Engineering, Feng Chia University, Taichung 40724, Taiwan; enchung@fcu.edu.tw; 2 Ph.D. Program of Electrical and Communications Engineering, Feng Chia University, Taichung 40724, Taiwan; 3 Department of Electrical Engineering, Feng Chia University, Taichung 40724, Taiwan; roy867542@gmail.com (C.-C.K.); wfliu@fcu.edu.tw (W.-F.L.)

**Keywords:** hydrophone, fiber Bragg grating, two-dimensional

## Abstract

This paper presents a high-sensitivity two-dimensional fiber-optic hydrophone designed for the detection and localization of underwater acoustic sources. The device comprises two sensing heads, each incorporating a fiber Bragg grating (FBG) embedded within a customized 3D-printed encapsulation. To enhance acoustic sensitivity, the design utilizes a silicone thin-film coupled with a pyramidal channel that spatially concentrates acoustic energy from the base to the apex, where the FBG is positioned. Incident acoustic pressure induces vibrations in the film, which are amplified by the channel structure, imparting strain on the FBG and resulting in a shift in the Bragg wavelength. The acoustic frequency response is demodulated by converting the overlapping optical power between the sensing and reference gratings into an electrical signal via a photodetector. By arranging the two sensing heads orthogonally, the system effectively determines the direction and angle of the acoustic source. Experimental results show a peak sensitivity of −210.59 dB re 1 V/μPa, with a FWHM of 57.92–66.27 Hz and a figure of merit (FOM) up to 3.64 dB/Hz. In addition, the acoustic-field SNR is approximately 26 dB in the dominant band, and the LOD is 64.19 dB re 1 μPa (10–400 Hz). Experimental validation confirms the hydrophone’s high sensitivity and localization accuracy, demonstrating its significant potential for underwater acoustic sensing applications.

## 1. Introduction

Fiber Bragg grating (FBG) is an important optical component in optical fiber sensing technology with periodic refractive index modulations along the fiber core axis to result in the reflected Bragg wavelength to be used for sensing the variations in temperature, strain, or pressure [1,2,3]. With the advantages of high sensitivity, compact size, immunity to electromagnetic interference, and distributed sensing capability, FBGs have been widely applied in structural health monitoring, vibration and pressure measurements, optical communications, and biomedical sensing, etc. and have increasingly extended to acoustic and underwater applications.

In underwater environments, conventional electronic sensors face critical limitations due to strong absorption of electromagnetic waves by water, along with susceptibility to signal interference and corrosion, which compromise durability and stability. These challenges highlight the advantages of FBG-based sensing technology. Early studies demonstrated the feasibility of FBG hydrophones [4,5,6,7]. Huang et al. improved sensitivity with an equivalent phase-shift design [8], while Lavrov et al. developed a thin FBG-based hydrophone array, enabling multipoint detection for ocean monitoring and communication [9]. Recent reviews have further highlighted the versatility of FBG acoustic sensing in seismic monitoring, structural inspection, and sound source localization [10,11,12].

In parallel, alternative optical and hybrid acoustic sensing technologies are rapidly emerging. McQueen et al. demonstrated a fiber-coupled photonic crystal hydrophone, exploiting nanophotonic structures for ultra-high-sensitivity and compact integration [13]. Buis et al. characterized a fiber laser hydrophone capable of detecting ultra-low-frequency acoustic signals from neutrino interactions, pushing optical hydrophones into astrophysical applications [14]. Kim et al. designed a wideband flextensional hydrophone, broadening frequency response via mechanical engineering principles [15]. Complementing these optical approaches, Wang et al. highlighted the rise of MEMS acoustic sensors, which leverage microfabrication for miniaturization, low power consumption, and scalable production, bridging the gap between research prototypes and real-world deployments [16]. The integration of MEMS with optical sensing, such as FBG or photonic structures, represents an exciting frontier for compact, multifunctional acoustic systems.

Meanwhile, FBG performance continues to benefit from material and structural innovations. Ahmad et al. presented a high-sensitivity pressure sensor using a thin metal diaphragm integrated with FBG [17], while Zhang et al. proposed helical long-period gratings for enhanced sensitivity [18]. Cross-disciplinary approaches have also combined FBGs with carbon-fiber composites [19] and novel acoustic transducers [20], further broadening their potential applications.

Despite these advances, challenges persist, including low-frequency sensitivity enhancement, long-term stability in harsh underwater environments, and robust signal processing for multipoint array systems. To address these issues, this study proposes a high-sensitivity, two-dimensional fiber-optic hydrophone that integrates a 3D-printed structural framework with a silicone membrane. Modeling and experimental validation confirm that the system can effectively detect and convert underwater acoustic signals into measurable electrical outputs while offering directional sensing capability.

In summary, the field of underwater acoustic sensing is rapidly advancing through diverse innovations, spanning FBG-based hydrophones, photonic crystal devices, fiber laser sensors, flextensional transducers, and MEMS-based systems. With continued progress in fiber fabrication, MEMS technology, and integrated photonics, these approaches are expected to converge, delivering stable, efficient, and multifunctional solutions for applications in ocean monitoring, underwater communication, neutrino detection, industrial safety, and defense systems.

## 2. Basic Principle and Sensing Head

### 2.1. Sensing-Head Fabrication

**Figure 1:**
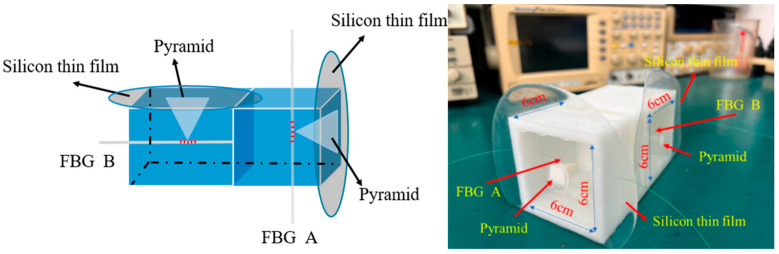
Hydrophone sensing head and physical diagram.

This fiber hydrophone is composed of sensing head A and sensing head B, as well as a 3D-printed housing structure, as shown in Figure 1. The structure of the sensing head is divided into two parts. The first part is the surface of receiving an acoustic signal consisting of a silicon thin-film to be boned with the base of a pyramid, which is located at the front end of the sensor for sensing the acoustic signals. The second part includes the sensing key component of the fiber Bragg grating and a 3D-printed housing structure. The fiber grating boned to the tip of a pyramid is pushed by the concentrated acoustic-wave force collected from the pyramid base area. Thus, the acoustic-wave force created from the first part is used for driving the fiber grating to result in the grating wavelength shift, which can be used for obtaining the acoustic-wave signals by combining the matched grating (or tunable laser) and photodetector. These two FBG sensing heads are respectively positioned at the *x*-axis and *y*-axis within the sensor structure for detecting the acoustic signals coming from different directions. The two FBGs employed in the sensing unit were fabricated in single-mode fibers by using a KrF excimer laser (248 nm) through a phase mask method with each grating length of approximately 2 cm. After the sensing heads were assembled, the sealant was applied to the structural gap around the sensing unit to prevent water from permeating to enhance the stability and durability of the sensor in underwater environments.

### 2.2. Fiber Bragg Grating Hydrophone Sensing Principle

The operating principle of this sensor is based on the grating wavelength shift due to the interaction pressure between acoustic waves and the silicon thin-film in the sensor. When the sound waves reach the sensor, the front-facing receiving membrane deforms due to the pressure exerted by the acoustic waves. This deformation can be represented by D [17]:

(1) $$D=\frac{Et^{3}}{12(1-\nu^{2})}$$

Equation (1) is used to describe the flexural rigidity of the membrane, which is a key parameter representing the membrane’s resistance to deformation. A thicker membrane or one with a higher elastic modulus results in a larger D value, indicating greater stiffness and reduced deformability. Here, $E_{D}$ denotes the Young’s modulus, t represents the membrane thickness, and ν is the Poisson’s ratio. The displacement of the membrane caused by vibration, denoted as $w\left(r\right)$, can be expressed using the following equation [17]:

(2) $$w\left(r\right)=\frac{pR^{4}{\left[1-{\left(\frac{r}{R}\right)}^{2}\right]}^{2}}{64D}$$

In this expression, R denotes the radius of the membrane, and p represents the magnitude of the acoustic pressure. When the membrane is excited by the incoming acoustic wave, it undergoes deformation and transfers the resulting dynamic force to the pyramid. The pyramid focuses this force and applies it directly to the fiber Bragg grating (FBG), causing it to be axially compressed and stretched. This process can be described by the following equation:

(3) $$\varepsilon =\frac{y}{L}$$

where, ε represents the axial strain induced in the FBG, y is the deformation transferred from the pyramid to the fiber, and L denotes the effective length of the FBG for strain sensing. The shift in the central wavelength of the grating is proportional to the mechanical strain applied to it and can be expressed by the following equation [17]:

(4) $$\Delta \lambda_{B}=\lambda_{B}\left(K_{\varepsilon }\varepsilon +K_{T}\Delta T\right)$$

In Equation (4), $\Delta \lambda_{B}$ represents the shift in the central wavelength of the fiber Bragg grating, and $\lambda_{B}$ is the initial central wavelength. The constants $K_{\varepsilon }$ and $K_{T}$ correspond to the strain and temperature sensitivities, respectively, and ΔT denotes the change in temperature. When the temperature is held constant, the influence of ΔT can be neglected, thereby simplifying the relationship between the wavelength shift and strain. The simplified expression is given as follows:

(5) $$\Delta \lambda_{B}=\lambda_{B}K_{\varepsilon }\varepsilon =\lambda_{B}K_{\varepsilon }\frac{y}{L}$$

### 2.3. Acoustic-Wave Frequency Demodulation

**Figure 2:**
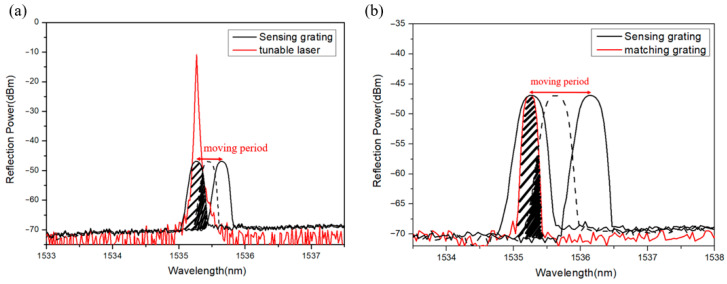
Overlapping spectrum: (**a**) the sensing grating and a tunable laser; (**b**) the sensing grating and the matched grating.

The demodulating acoustic-frequency mechanism of grating wavelength shift is based on the overlapping reflection power between the sensing grating and a narrowband light source (such as a tunable laser or a broadband source combined with a narrowband filter). For the sensing head of FBG A, the overlapping reflection spectra created by the sensing grating and the output spectrum of a tunable laser are shown in Figure 2a for demodulating the testing acoustic-wave frequency. This is due to the fact that the output light of a tunable laser is used for a narrowband light source to be transmitted into the fiber circulator and then is launched into the sensing fiber grating to get the overlapping power with the sensing grating spectrum. When this sensor senses the tested acoustic signals, the grating wavelength will be shifted to result in the variation of overlapping optical power, which is input to a photodetector for obtaining the time function of the input optical overlapping power. Similarly, for the sensing head of FBG B, a narrowband light source is composed of an ASE broadband light source, a fiber coupler and a matched FBG, whose function is similar to a tunable laser. Figure 2b shows the overlapping optical spectra between the matching grating and sensing grating B, which is sent to the photodetector (InGaAs) for converting the overlapping optical power into an electrical signal to be shown in an oscilloscope. By using this process, the amplitude and time function of the electrical signal can be displayed on the oscilloscope for further analysis of the tested acoustic waves. In addition, for obtaining the nice sensing performance, the wavelength of the tunable laser is tuned to match the grating wavelength of the FBG A sensing head. Similarly, the center wavelength of the matching fiber grating and the sensing fiber grating (FBG B) must initially be matched because the overlapping optical spectra transmitted to the photoelectric converter will be obtained according to the overlapping power of the sensing center wavelength and the matching center wavelength. This optical frequency-demodulation design investigates the performance response of the sensor to underwater acoustic waves.

## 3. Experimental Setup and Results

### 3.1. Experimental Configuration

**Figure 3:**
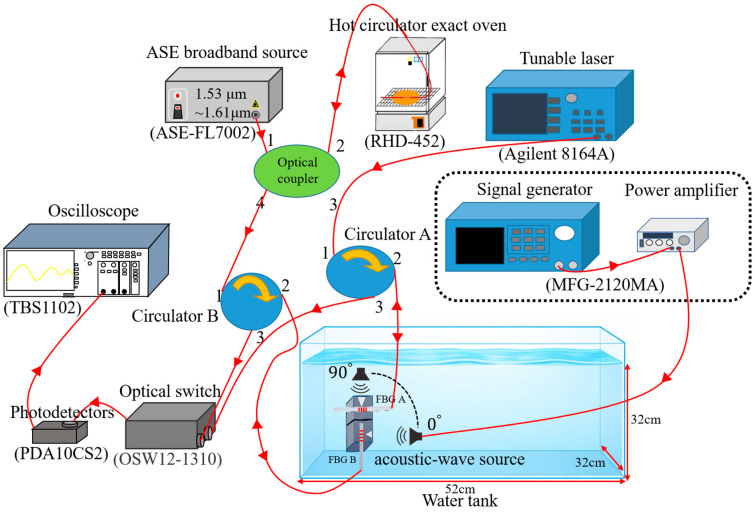
Hydrophone experimental setup.

The experimental setup is schematically illustrated in Figure 3. Acoustic waves are generated using a loudspeaker submerged in a water tank, driven by a signal generator via a power amplifier (MFG-2120MA, GW Instek, New Taipei City, Taiwan; used to provide the driving signal and amplification for the loudspeaker). The vibration of the loudspeaker diaphragm induces fluid displacement to generate acoustic waves that propagate along the axis of vibration. The amplitude and frequency of these waves are controlled by the output power of the amplifier and the setting frequency of the signal generator, respectively. To accommodate the near-field characteristics of the low-intensity acoustic waves, a water tank with dimensions of 52 cm × 32 cm × 32 cm is employed for simulating near-field measurement conditions analogous to electromagnetic wave analysis. The fiber hydrophone sensor is fixed within the tank, positioned directly facing the acoustic source. Incident acoustic waves propagate through the water and impinge upon the sensor’s frontal membrane. The resulting acoustic force is transmitted through the pyramid structure to the fiber Bragg gratings (SMF-28, Corning, Inc., Corning, NY, USA) to induce the grating wavelength shift. This grating wavelength shift alters the overlapping optical power between the sensing grating and a matched grating (SMF-28, Corning, Inc., Corning, NY, USA) with tunable laser (Keysight Technologies, Inc., Santa Rosa, CA, USA), which is subsequently converted into electrical signals via a photodetector (PDA10CS2, Thorlabs, Inc., Newton, NJ, USA); converting optical power variations into voltage signals). These signals are monitored on an oscilloscope (TBS1102, Tektronix, Beaverton, OR, USA) for analyzing the sensor’s sensitivity and frequency response. As shown in the optical path diagram in Figure 3, light from a tunable laser (Agilent 8164A, Santa Clara, CA, USA; interrogation source for the FBG A channel) is directed to circulator A. The light enters port 1 and exits through port 2 toward the sensing head FBG A. The reflected optical signal, containing the overlapping power information to be returned to port 2 is directed to port 3, and subsequently transmits through an optical switch (OSW12-1310, Thorlabs, Inc., Newton, NJ, USA; used for channel routing/selection) to the photodetector (PDA10CS2). Similarly, light from an amplified spontaneous emission (ASE) broadband source (ASE-FL7002, Thorlabs, Inc., Newton, NJ, USA; broadband source for the matched-grating-based demodulation in the FBG B channel) is launched into a matched grating via an optical coupler (the matched grating is placed in a hot circulator exact oven (RHD-452, Chuanhua Precision, Taipei City, Taiwan) to thermally tune its reflection spectrum to match the sensing grating). The reflected light enters circulator B through port 4 and exits via port 2 to the sensing head FBG B. The overlapping optical power between the matched grating reflection and the FBG B spectrum returns to port 2, exits through port 3, and is routed to the optical switch. The final optical signals are detected by photodetectors (PDA10CS2) with the corresponding electrical signals visualized and analyzed using an oscilloscope (TBS1102).

**Figure 4:**
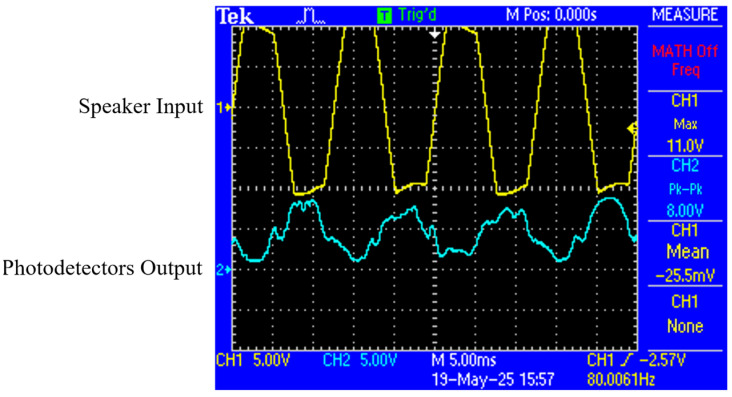
Time-domain comparison between speaker input and photodetector output.

To confirm the conversion between the input signal (e.g., acoustic wave) of the loudspeaker and the output signal of the photodetector, the time-domain signals driving the loudspeaker and the output of the photodetector are synchronously shown in Figure 4, in which we can see that the photodetector accurately detects the acoustic waveform with a phase shift of 180 degrees.

### 3.2. Spectrum of Acoustic-Wave Source

**Figure 5:**
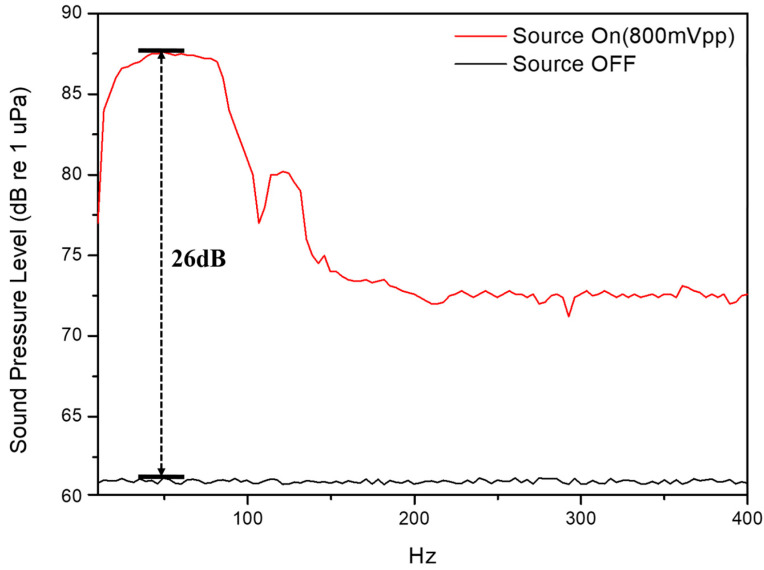
Frequency response of the loudspeaker from 10 Hz to 400 Hz.

A simple acoustic-wave source, as shown in Figure 3, is generated by a speaker combined with a signal generator and a power amplifier. Before the fiber hydrophone detection experiment, it is necessary to confirm the underwater acoustic spectrum for measurement characterization. Therefore, a sound-level meter equipped with a suitable underwater sensing probe, with a frequency response of 5 Hz–5 kHz, is used as a detector to monitor the acoustic-wave level and record the sound pressure level (SPL, dB re 1 μPa) at different frequencies. The experimental results are shown in Figure 5, where the frequency response exhibits a dominant band from 25 to 90 Hz with a non-uniform output response. Thus, this original acoustic spectrum serves as the reference standard and baseline control for subsequent experiments to ensure measurement accuracy. In addition, Figure 5 shows the source-off baseline for allowing the acoustic-field SNR to be directly evaluated from the SPL gap between the source-on and source-off spectra. As indicated in Figure 5, the SNR is approximately 26 dB in the dominant band (25–90 Hz).

The sound-level meter readings were calibrated/validated using a standard reference hydrophone (Brüel & Kjær Type 8103, HBK World, Virum, Denmark). Therefore, the SPL values measured in Figure 5 are used as the pressure reference for converting the subsequent results into sensitivity. The detected voltage is first converted from peak-to-peak to RMS as follows:

(6) $$V_{rms}(f)=\frac{V_{pp}\left(f\right)}{2\surd 2}$$

The corresponding acoustic pressure is obtained from the SPL as:

(7) $$p_{rms}(f)(\mu Pa)=10^{SPL(f)/20}$$

Accordingly, the voltage-to-pressure sensitivity is calculated by:

(8) $$S(f)=20{log}_{10}(\frac{V_{rms}(f)}{p_{rms}(f)})\left[dB\;re\;1\;V/uPa\right]$$

Because the measured voltage is obtained from the output of photo-receiver amplification, the reported sensitivity is further compensated by subtracting the receiver gain setting and the minimum trans-impedance conversion under Hi-Z termination, i.e.,

(9) $$S_{comp}\left(f\right)=S\left(f\right)-G_{PD}-20{log}_{10}(Z_{t,min})$$

In this work, $G_{PD}$=70 dB and $Z_{t,min}$= 1.51 × 103 V/A, which corresponds to $20{log}_{10}(Z_{t,min})$ = 63.58 dB. Using the equations from (6) to (9), all subsequent figures have been converted and reported as the compensated sensitivity in dB re 1 V/μPa. For each tested condition, the measurement was repeated five times. The reported sensitivity values are presented as the mean, and repeatability is quantified by the standard deviation, with around 0.7 dB (in dB re 1 V/μPa) under the same experimental configuration. In addition, the limit of detection (LOD) is defined as the minimum SPL exceeding the source-off (static-water) baseline by 3 dB to yield an LOD of 64.19 dB re 1 μPa. The operating bandwidth (FWHM) is defined as the continuous frequency interval (from F_low_ to F_high_) within the measured range, where the compensated sensitivity remains within 3 dB of the peak value, ${FWHM=F}_{high}-F_{low}$.

### 3.3. Directional Discrimination of Acoustic Sources

**Figure 6:**
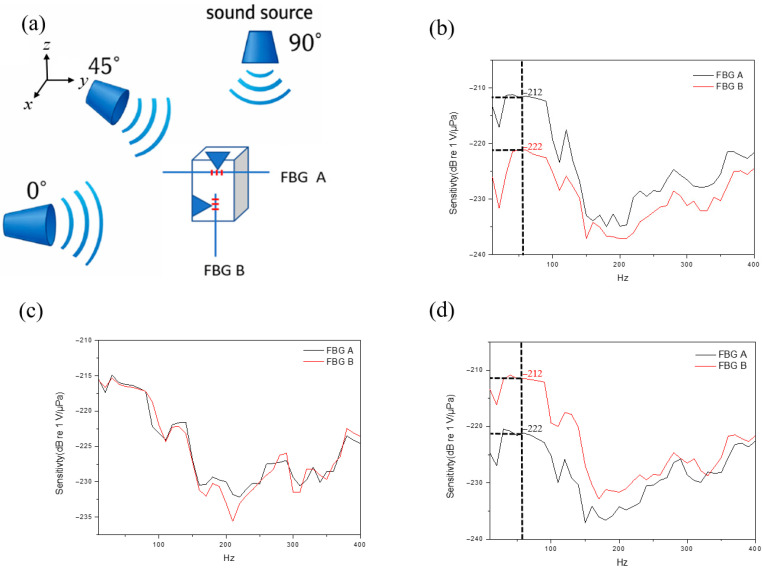
(**a**) Schematic diagram illustrating the relative positions between the acoustic signal and the sensors. (**b**) Frequency responses of FBG A and FBG B to the acoustic signal at an incident angle of 90°. (**c**) Frequency responses of FBG A and FBG B to the acoustic signal at an incident angle of 45°. (**d**) Frequency responses of FBG A and FBG B to the acoustic signal at an incident angle of 0°.

To investigate the directionality of the acoustic source, the acoustic signal was respectively positioned at three distinct angles relative to the sensors, including 90°, 45°, and 0°, as illustrated in Figure 6a. Figure 6b–d depict the measured acoustic-signal spectra of the sensors corresponding to the three different angles. The fluctuation of measured spectra is due to the signal output power of acoustic-source and tank-reflection effects. For the acoustic source to be located at an angle of 90°, the detecting level for the sensing head FBG A is evidently higher than that of FBG B, as shown in Figure 6b. For the angle of 45°, the detecting level of FBG A is almost identical to that of FBG B, as shown in Figure 6c. For the angle of 0°, the detecting level of FBG A is obviously lower than that of FBG B, as shown in Figure 6d. Thus, the direction of the sound source can be estimated from the detecting level of both FBG A and FBG B. For example, by giving the acoustic-source signal with the frequency of 60 Hz, the source direction from 0° to 90° can be distinguished in the range from the data (−222 dB re 1 V/μPa, −212 dB re 1 V/μPa) to the data (−212 dB re 1 V/μPa, −222 dB re 1 V/μPa), as shown in the dash line of Figure 6d and Figure 6b respectively. As the data of three angles were only measured in this study, the results are mainly intended to verify the trend of angle-dependent response and the feasibility of directional discrimination. In future work, a polar pattern will be established through finer angular sweeps, and sound-source bearing estimation and localization methods will be further developed accordingly.

### 3.4. Effect of Pyramid Base Area

**Figure 7:**
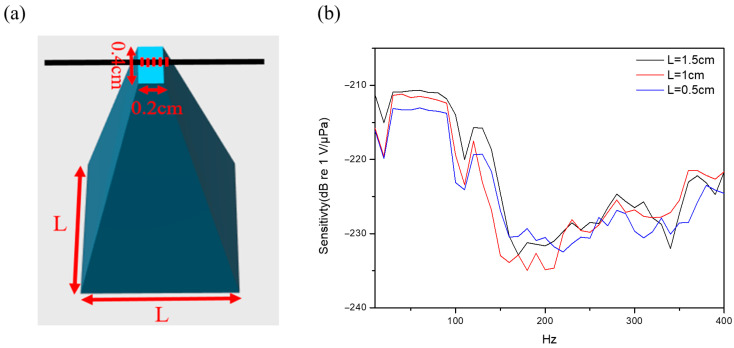
(**a**) Schematic diagram illustrating the different pyramid base dimensions. (**b**) Frequency responses versus different pyramid base dimensions in the frequency range from 10 Hz to 400 Hz.

To empirically evaluate sensing performance, key design parameters are modified and compared through comparative experimental analysis. Acoustic waves from the source initially impinge upon the thin film and are transmitted to the pyramid structure. The pyramid can concentrate the acoustic force at its tip, where it exerts a stress on the FBG, as illustrated in Figure 7a. During the experiments, the pyramid tip was firmly pressed into close contact with the grating region to ensure efficient optical coupling. The geometric and material parameters of the pyramids used in this study are summarized in Table 1. For investigating the influence of the pyramid’s base area on sensing performance, the base dimensions are varied while maintaining a fixed height of 1.8 cm and a constant source-to-sensor distance of 2 cm. Three distinct base dimensions are employed for the measurements. The experimental results, as shown in Figure 7b, demonstrate a clear response within the frequency range from 45 Hz to 90 Hz. As indicated in the figure, the pyramid with the largest base area, 1.5 cm × 1.5 cm, exhibits the highest sensing intensity, achieving the highest sensitivity of −210.66 dB re 1 V/μPa at f = 60 Hz. This behavior is attributed to the larger base area’s capacity to collect greater acoustic power, thereby concentrating more energy at the pyramid tip. The pyramid with a 1.0 cm × 1.0 cm base area demonstrates an intermediate performance (−211.17 dB re 1 V/μPa), whereas the smallest base area (0.5 cm × 0.5 cm) yields the lowest sensitivity (−213.03 dB re 1 V/μPa). These results indicate that, within the tested range and under the present experimental configuration, the measured sensitivity tends to increase with the pyramid base area.

### 3.5. Effect of Structural Aperture Size

**Figure 8:**
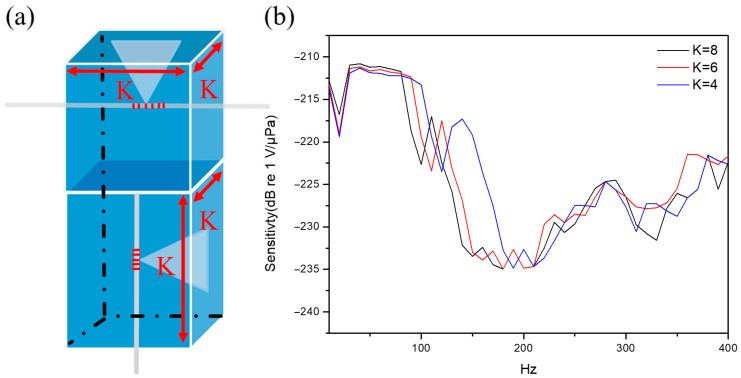
(**a**) Schematic diagram illustrating the different aperture K sizes of the structural design. (**b**) Frequency responses of three different aperture sizes in the frequency range from 10 to 400 Hz.

To evaluate the effect of sensing spectrum, the aperture size within the hydrophone structure is changed for the aperture dimension K from 4 to 8 cm, as illustrated in Figure 8a. The experimental results are shown in Figure 8b with three different K sizes, including 4 cm, 6 cm, and 8 cm, to obtain the maximum sensitivities of −211.32 dB re 1 V/μPa, −211.17 dB re 1 V/μPa, and −210.81 dB re 1 V/μPa, respectively. This fact demonstrates a positive correlation between the maximum sensitivity and the aperture dimension. Notably, while the hydrophone with the smallest aperture exhibits a frequency response shift toward the higher-frequency spectrum, it indicates a better high-frequency response. But its maximum sensitivity is significantly lower than that of the other sizes. This phenomenon is intrinsically attributed to the reduced acoustic force exerted on the sensing membrane by a smaller aperture. Consequently, the conversion from mechanical energy into electrical signals is diminished. Although a smaller aperture has a wider frequency response, the reduction in driving acoustic force limits the overall signal intensity.

**Figure 9:**
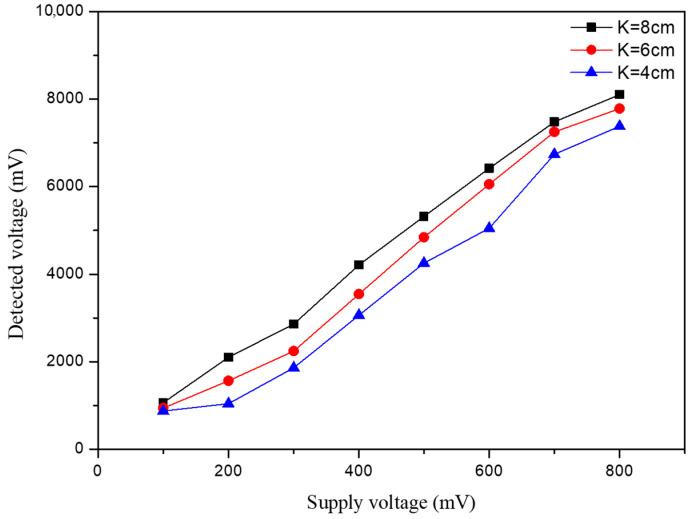
Detected voltage curves for the structure aperture size versus the supply voltage.

To further examine the sensing response, measurements were conducted on different supply voltages of the signal generator for three aperture sizes. As illustrated in Figure 9, the hydrophone with an aperture size of K (8 cm) exhibits the highest detected voltage (mV) due to its maximized effective sensing area. As the aperture size K is decreased from 6 cm to 4 cm, the detected voltage is reduced significantly to exhibit a clear downward trend.

### 3.6. The Elasticity Effect of Silicon Thin-Film

**Figure 10:**
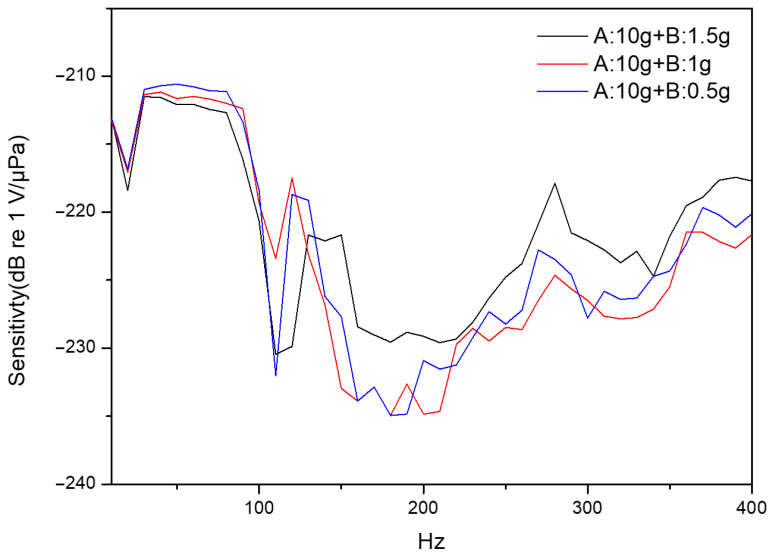
Frequency responses of different thin-film elasticities in the frequency range from 10 to 400 Hz.

A liquid silicone rubber (SL2501 A and SL2501B, KCC Corporation, Seoul, South Korea) including two different components A and B was used for the silicone thin-film. Component A is for the silicone base, and component B is for the curing agent. The silicone thin-film is fabricated by mixing component A with component B, curing in an appropriate ratio. The concentration of the curing agent plays a pivotal role in determining the mechanical properties of the resulting thin-film. A spin-coating facility with a fixed rotational speed is employed to ensure the uniform thin-film thickness. Three different specimens are prepared, including component A to be fixed at 10 g to change component B’s weight to 1.5 g, 1 g, and 0.5 g, respectively. All silicon thin-films are fabricated under identical processing conditions. Under the same spin-coating condition, the measured thicknesses of the silicone thin-films were 0.88 mm, 0.91 mm, and 0.95 mm, corresponding to the curing agent weights of 0.5 g, 1.0 g, and 1.5 g, respectively. Due to the thickness variation among these samples being rather small, the performance difference is mainly attributed to the change in elasticity induced by the curing agent concentration. Subsequently, the experimental results of frequency response are conducted to evaluate the effect of mechanical characteristics on sensitivity and stability. Figure 10 shows that the optimal frequency response by using the thin-film fabricated with 0.5 g of curing agent e to achieve a maximum sensitivity of −210.59 dB re 1 V/μPa. In comparison, the thin-films prepared with 1 g and 1.5 g of curing agent demonstrate the reduced sensitivities of −211.17 dB re 1 V/μPa and −211.5 dB re 1 V/μPa, respectively. This fact indicates that increasing the curing agent content enhances the thin-film stiffness, which restricts vibration amplitude and degrades frequency response. Thus, the 0.5 g formulation demonstrates superior sensitivity and stability to offer a distinct advantage in enhancing overall sensing performance.

### 3.7. Structural Internal Pressure Effect

**Figure 11:**
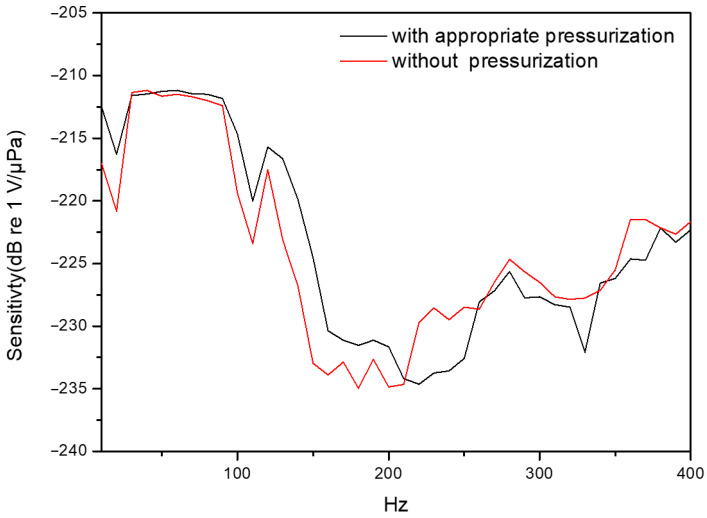
Frequency response of the hydrophone structure in different internal pressures.

For investigating the effect of structural-cavity internal pressure, the structural cavity is appropriately pressured in order to obtain better sensing performance. This is due to the fact that the ambient hydrostatic pressure will induce an inward deformation of the diaphragm of the sensor, which affects sensing sensitivity. After the structural cavity is appropriately pressured, the cavity-internal pressure is balanced with the external hydrostatic pressure to obtain a larger vibration response under acoustic excitations. The frequency response of the sensor in the structural cavity with and without pressurization is shown in Figure 11. From this figure, we obviously see that senor pressurization will improve sensing performance.

### 3.8. Effect of Propagation Distance

**Figure 12:**
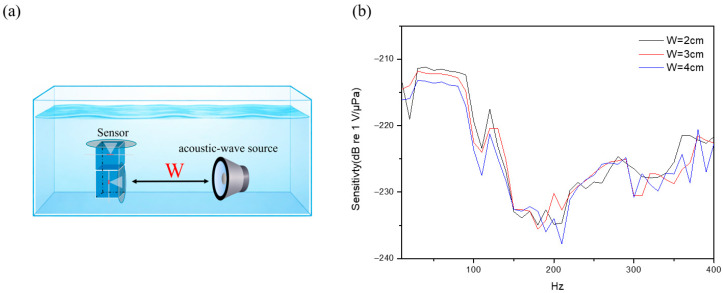
(**a**) Schematic diagram illustrating the separation distance between the sensor and the acoustic source. (**b**) Frequency responses of the structure at varying distances over the frequency range of 10 Hz to 400 Hz.

To investigate the influence of acoustic-signal propagation distance, the separation between the acoustic source and the sensor is systematically set to 2 cm, 3 cm, and 4 cm, as illustrated in Figure 12a. By means of a comparative analysis of frequency response variations, the attenuation and transmission efficiency can be obtained due to the acoustic waves propagating through a medium to cause attenuation and scattering. From Figure 12b, we can see that the maximum sensitivity measured by the sensor is inversely proportional to the distance. At the separation of 2 cm, 3 cm, and 4 cm, the maximum sensitivities of −211.17 dB re 1 V/μPa, −211.76 dB re 1 V/μPa, and −213.14 dB re 1 V/μPa are respectively measured. The fact indicates that the acoustic propagation is governed by multiple loss factors, leading to gradual energy attenuation. These factors include absorption loss (conversion of acoustic energy to heat), scattering loss (deviation caused by inhomogeneities), and viscous loss (dissipation due to fluid viscosity).

### 3.9. Overall Summary and Design Guideline

Table 2 summarizes the best-performing condition identified among the tested levels from each one-factor-at-a-time parametric study. Overall, the proposed hydrophone shows its strongest and most repeatable response in the low-frequency region, and the extracted cases provide both high peak sensitivity and FWHM bandwidth. To further quantify the trade-off between peak sensitivity and effective bandwidth, a figure of merit (FOM) is defined as the ratio of the absolute peak sensitivity to the corresponding bandwidth (FWHM). Based on the best-performing extracted cases in Table 2, the calculated FOM ranges from 3.19 to 3.64 dB/Hz. Although the thin-film elasticity case provides the highest peak sensitivity, the aperture-size configuration yields the highest FOM owing to its relatively narrower −3 dB bandwidth. These observations indicate that, within the tested parameter ranges, adjusting the structural aperture can concentrate the acoustic response within the effective operating band while maintaining competitive peak sensitivity.

## 4. Conclusions

This paper experimentally demonstrates a fiber Bragg grating (FBG) hydrophone capable of simultaneous acoustic-wave detection and source localization. Attributed to the design of a dual-sensing-head architecture, the experimental results reveal significant discrepancies in the frequency responses of the two sensing heads as the angle of the acoustic source varies, thereby validating the sensor’s directional discrimination capability. To obtain better sensing performance, this study conducted a systematic investigation of changing key structural parameters, including the pyramid base area, internal pressure, aperture size, and membrane elasticity. Within the tested parameter ranges, each design factor demonstrated a nice operating condition, and its impact on the frequency response and sensitivity was experimentally quantified. The FWHM and FOM are summarized in Table 2 as an empirical reference for future design refinement and system integration. Collectively, these experimentally identified parameter settings effectively enhance sensing stability and resolution to establish a robust empirical foundation for the advancement of high-performance underwater acoustic sensing technologies.

## Figures and Tables

**Table 1:** Pyramid parameters.

Parameter	Value	Unit
Pyramid height	18	mm
Base side length	0.5/1.0/1.5	cm
Tip area	0.08	cm ^2^
Wall inclination angle (0.4 cm side, 2 faces)	88/81/74	deg
Wall inclination angle (0.2 cm side, 2 faces)	85/78/71	deg
Material	ABS resin	

**Table 2:** Summary of the nice parameter configurations obtained from each parametric analysis with the corresponding peak sensitivity (S_peak_), full width at half maximum (FWHM), and figure of merit (FOM).

Study Factor	Condition	$S_{peak}$ (dB)	$F_{low}$ (Hz)	$F_{high}$ (Hz)	FWHM	FOM
Effect of pyramid base area	L = 1.5 cm	−210.66	25.00	90	65	3.24
Effect of structural aperture size	K = 8	−210.81	25.10	83.02	57.92	3.64
The Elasticity effect of silicon thin-film	A:10 g + B:0.5 g	−210.59	25.55	90.45	64.90	3.24
Structural internal pressure effect	with appropriate pressurization	−211.18	25.00	90.00	65.00	3.25
Effect of propagation distance	W = 2 cm	−211.17	26.32	92.59	66.27	3.19

## Data Availability

The original contributions presented in this study are included in the article. Further inquiries can be directed to the corresponding author.
